# Combination of venetoclax with CHG regimen in refractory/relapsed T-lymphoblastic lymphoma/acute lymphoblastic leukemia: a case series and literature review

**DOI:** 10.1007/s12672-025-03055-4

**Published:** 2025-07-01

**Authors:** Mei Zhou, Yuze Yang, Xiaoyan Zhang, Yijie Jiao, Zhenxing Guo

**Affiliations:** Department of Hematology/Oncology, First Hospital of Tsinghua University, School of Medicine, Tsinghua University, Beijing, 100016 PR China

**Keywords:** T lymphoblastic lymphoma/T acute lymphoblastic leukemia, Venetoclax, Homoharringtonine, CHG regimen, Refractory/relapsed

## Abstract

T lymphoblastic lymphoma/acute lymphoblastic leukemia (T-LBL/ALL) is a highly aggressive hematologic malignancy, with particularly poor outcomes for refractory/relapsed (R/R) patients. This article reports the efficacy and safety of V-CHG regimen (Venetoclax, cytarabine, homoharringtonine, G-CSF) in the treatment of three R/R T-LBL/ALL cases. One 69 years old female patient with T-ALL experienced a twice relapse and achieved morphological complete remission (CR) with one cycle of V-CHG regimen. Another 65 years old female patient with T-LBL progressed to T-ALL after continuous CR for 10 months and failed by two different salvage chemotherapy regimens. She achieved minimal residual disease (MRD) negative CR with a third-line treatment based on V-CHG regimen. The third T-ALL patient was a 26 years old male who resistant to VDCP induction regimen and achieved morphological CR with one cycle of V-CHG regimen, and obtained MRD negative CR after second cycle treatment. All the three patients were well tolerated to the V-CHG regimen. The results of this study indicate that the V-CHG regimen is effective and safe for R/R T-LBL/ALL, warranting further application in the future.

## Introduction

T lymphoblastic lymphoma/acute lymphoblastic leukemia (T-LBL/ALL) is a highly aggressive malignant tumor that originates from immature precursor T cells [1]. Historically, the complete remission (CR) rate for T-ALL has been between 70% and 90%, with a 3–5 year disease-free survival rate ranging from 30 to 60% [2]. Despite great improvements have been achieved in treatment for T-ALL patients due to the application of targeted drugs, monoclonal antibodies, chimeric antigen receptor (CAR) T cells therapy, allogeneic hematopoietic cell transplantation(allo-HSCT) techniques and various supportive strategies [3], 5–10% of patients remain primary refractory, and 30–60% encounter relapse [4]. Consequently, the treatment of relapsed/refractory (R/R) T-LBL/ALL patients remains challenging due to the lack of effective therapeutic options.

Venetoclax (VEN) has shown good efficacy in the treatment of acute myeloid leukemia (AML) [5, 6], but reports on T-LBL/ALL are still limited. Similarly, homoharringtonine (HHT), while playing an important role in the treatment of myeloid leukemia, has scarcely been used for ALL. Here, we present three adult patients with R/R T-LBL/ALL who were successfully salvaged with V-CHG regimen. These preliminary but promising results might lay the foundation for further basic research and clinical trials to explore the potential of the V-CHG regimen in the treatment of R/R T-ALL patients.

## Case presentation

### Case 1

A 69-year-old female patient presented with dizziness, fatigue and pallor in September 2020. The complete blood count revealed WBC 2.15 × 10^9^/L, HB 91 g/L and PLT 107 × 10^9^/L. Morphology evaluation revealed 78% blast cells in bone marrow (BM). Flow cytometry (FCM) analysis showed 69.38% blast cells, which was characterized by cCD3^+^, CD99^+^, TDT^+^, CD7Bright^+^, CD5^+^, CD33^+^, CD3^+−^, CD13^+−^, CD117^+−^, CD2^−^, CD4^−^, CD8^−^, CD1a^−^, CD10^−^, CD56^−^, CD11b^−^, CD123^−^, CD19^−^, CD22^−^, CD15^−^, CD14^−^, CD64^−^, HLA-DR^−^, cCD79a^−^ and MPO^−^. The cytogenetics and molecular biology results were shown at Table 1. The patient was diagnosed as T-ALL and achieved CR by induction chemotherapy with two cycles of VDCP regimen (Vindesine, Daunorubicin, Cytarabine and Prednisone). Subsequently, she underwent two cycles of the VDCP, and one cycle of intermediate-dose Cytarabine (ID- Ara-C) for intensive treatment with continuous CR. Due to poor adherence, the patient didn’t proceed with further treatment. Unfortunately, she relapsed in February 2022 and VDCP re-induction failed. Subsequently, she achieved a short-term (two months) CR by ID- Ara-C but relapsed again in August 2022. Both ID- Ara-C and V-CEOP re-induction failed. Therefore, the patient treated with V-CHG regimen (VEN 100 mg d1-d10, Homoharringtonine (HHT) 1 mg d1-d10, Ara-C 15 mg subcutaneously q12h d1-d10, G-CSF 300 µg/d from day 0 until WBC count > 20 × 10^9^/L) for one cycle in June 2023, and achieved morphology CR for 1 month without any medication treatment. Even restart of V-CHG regimen after relapse, the patient still achieved CR for 1 month again (Table 2). Finally, the patient participated in a CD7 CAR-T clinical trial. The adverse events were showed in Table 3.

**Table 1:** Baseline patient characteristics

Characteristics	P1	P2	P3
Age/gender	69/F	65/F	26/M
WBC (×10^9^/L)	2.15	10.6	43.4
HB (g/L)	91	113	140
PLT (×10^9^/L)	107	169	102
Blast cells in BM (%)	78	–	93
Immunophenotype	T-ALL	T-LBL	T-ALL
Karyotype	46,XX, add(2)(q27)[5]/46,XX[15]	46, XX, add (7) (p15) [5] /46, XX [15]	46, XY, add (1) (p34), add (2) (p21), add (6) (p23), -13, +mar, inc [cp6]/46, XY [14]
Fusion gene	(–)	(–)	(–)
Gene mutation	ETV6	NRAS, DNMT3A, ETV6	JAK1, JAK3, NOTCH1, ARID1A, PHF6
Blast cells before V-CHG reinduction (%)	90	55	81.5
Disease status before V-CHG reinduction	Relapsed	Relapsed	Refractory
Prior lines of treatment	4	3	1

**Table 2:** Response for patients who received V-CHG

Characteristics	P1	P2	P3
Cycles of reinduction by V-CHG regimen/result/MRD Outcome	1/CR/ 1.01 × 10^−2^	1/CR/ <1 × 10^−4^	1/CR/8 × 10^−4^ 2/CR/<1 × 10^−6^
	Maintain CR for 1 month after discontinuation, CR again when reuse	In continuous CR for 4 months till relapse	In continuous CR for 11 months till now

**Table 3:** Adverse events

Adverse events	P1	P2	P3
Anemia/grade/duration	Yes/III/12d	Yes/III/20d	Yes/III/21d
Neutropenia/duration	Yes/11d	Yes/10d	No
Thrombocytopenia/duration	Yes/11d	Yes/15d	No
Febrile neutropenia	Yes	No	No
Nausea and vomiting/grade	No	No	Yes (III)

Anemia(gradeIII): HB < 80 g/L; Neutropenia: NEUT < 0.5 × 10^9^/L; Thrombocytopenia: PLT < 20 × 10^9^/L

### Case 2

A 65-year-old female presented with enlarged lymph nodes in July 2022. The complete blood count revealed WBC 10.6 × 10^9^/L, HB 91 g/L and PLT 107 × 10^9^/L. Bone marrow aspiration result suggested CMML, and the pathological biopsy of the right axillary lymph node indicated lymphoid hematopoietic system immature cell tumor. Further examination by FCM showed 31.15% blasts in lymph node, which characterized by CD34^+^/CD45dim, cCD3^+^, CD5^+^, CD7^+^, TdT^+^, CD56^+^, CD33^+^, CD38^+^, CD13^+−^, CD123^+−^, HLA-DR^+−^, cMPO^−^, cCD79a^−^, CD2^−^, CD3^−^, CD4^−^, CD8^−^, CD1a^−^, CD16^−^, CD117^−^, CD11b^−^, CD15^−^, CD64^−^, CD14^−^, CD36^−^, CD300e^−^, CD10^−^, CD20^−^ and CD19^−^. The cytogenetics and molecular biology results were showed at Table 1, and tyrosine kinase fusion genes were negative detected by multiple fluorescence quantitative PCR. Therefore, the patient was diagnosed as T-LBL (IIIB, low to intermediate risk), combined with CMML (classified as CMML-1 by WHO). She achieved partial remission with 2 cycles of VDCP regimen. Subsequently, she achieved CR by intensive chemotherapy with one cycle of ID Ara-C, COAP regimen (Cyclophosphamide, Vindesine, Cytarabine, Dexamethasone), two cycles of high-dose Methotrexate (HD-MTX) and one cycle of VDCP. Then she achieved continuous CR for 10 months by maintenance therapy with the VMMP regimen (Vindesine, 6-MP, MTX, Prednisone). However, morphology showed 2.5% blast lymphocytes in BM in March 2024. She failed with VDCP and ID-Ara-C regimen for re-induction, subsequent morphology of BM showed 55% blast lymphocytes. Finally, in June 2024, the patient achieved CR with FCM MRD negative (1 × 10^−4^) with a cycle of the V-CHG + Btz regimen (Ven 100 mg d1, 200 mg d2, 400 mg d3-10, Ara-C 12.5 mg every 12 h d1-d10, HHT 1 mg d1-10, G-CSF 300 µg/d from day 0 until WBC count > 20 × 10^9^/L, Bortezomib 1.5 mg day 1 and 8) and kept continuous CR for up to 4 months until relapse again (Table 2).

**Fig. 1:**
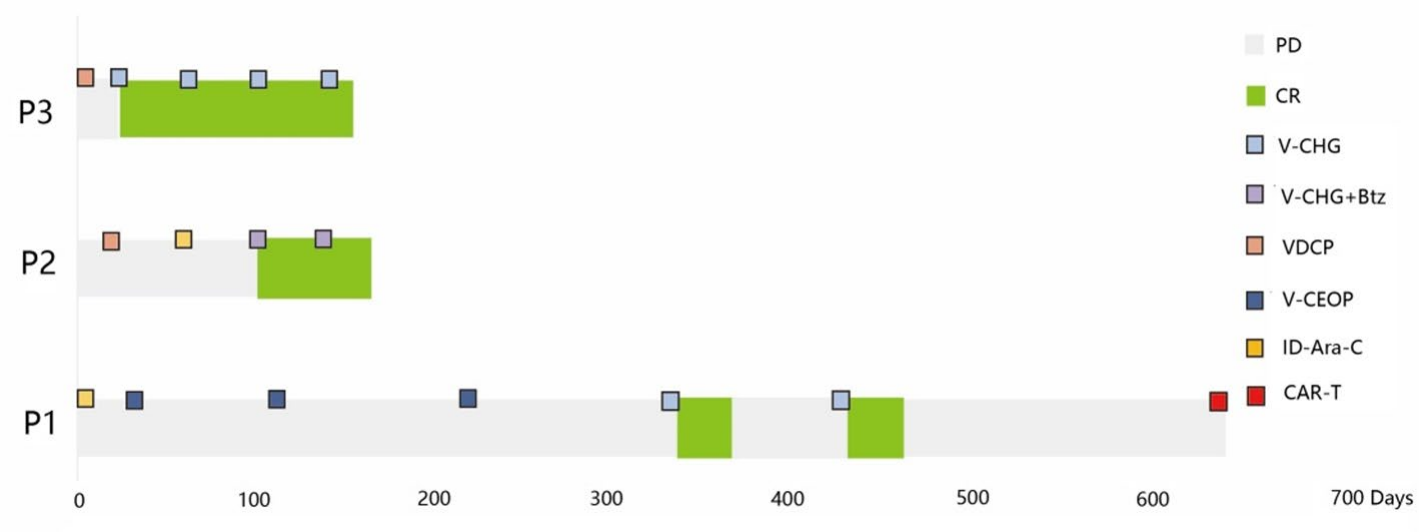
Dynamics of response to regimen after refractory/relapsed. *PD* progressive disease, *CR* complete remission, *VDCP* Vindesine, Daunorubicin, Cytarabine and Prednisone, *ID-AraC* intermediate-dose Cytarabine, *V-CEOP* Venetoclax, Cyclophosphamide, Etoposide, Vindesine, Dexamethasone, *V-CHG* Venetoclax, Cytarabine, HHT, G-CSF, *V-CHG + Btz* Venetoclax, Cytarabine, HHT, G-CSF, Bortezomib

### Case 3

A 26-year-old male presented with enlarged lymph nodes, low grade fever and night sweats in April 2024. The complete blood count revealed WBC 43.40 × 10^9^/L, HB 140 g/L and PLT 102 × 10^9^/L. Morphology revealed 93% blast cells in BM. FCM showed 91.42% blasts in BM, which was characterized by CD7^+^/CD45dim^+^, CD5dim^+^(with 85% positive), CD99^+^, CD48dim^+^, CD38^+^, CD117^+^, cCD3^+^, cTdT^+^, CD34^+−^, CD13^+−^, CD2^−^, CD3^−^, CD4^−^, CD8^−^, CD1a^−^, CD56^−^, CD10^−^, CD19^−^, CD22^−^, HLA-DR^−^, CD11b^−^, CD15^−^, CD33^−^, CD14^−^, CD36^−^, cMPO^−^ and cCD79a^−^. The cytogenetics and molecular biology results were showed at Table 1. Therefore, the patient was diagnosed with T-ALL. Unfortunately, he failed by induction chemotherapy of VDCP regimen and morphology evaluation revealed 81.5% blast cells in BM on the 14th day. What ‘s more, the patient developed acute pulmonary embolism at that time. Thus, the V-CHG regimen was applied from the 15th day (VEN 100 mg d1, 200 mg d2, 400 mg d3–12, Ara-C 20 mg every 12 h d1-12, HHT 2 mg d1-12, and G-CSF 300 µg/d from day 0 until WBC count > 20 × 10^9^/L). Surprisingly, morphology showed 1% blast cells in BM on the 8th day for re-induction with the V-CHG regimen. FCM showed 0.08% blast cells on the 28th day. Furthermore, chromosomes and gene mutation turned to normal level. Subsequently, he achieved MRD negative (< 1 × 10^−6^) CR after the second cycle of V-CHG regime and continuous CR for 11 months up to now (Table 2).

## Discussion

R/R T-LBL/ALL patients typically have a poor prognosis [7], with a CR rate ranging between 20 and 40% [4]. VEN, an effective BCL-2 inhibitor primarily targeting AML, has shown promise in treating T-ALL as well. Fundamental studies have demonstrated that T-ALL was sensitive to VEN in vitro [8, 9], and this sensitivity was closely related to T cell differentiation stage [10, 11]. Furthermore, not only in refractory early T-cell precursor acute lymphoblastic leukemia (ETP-ALL), but also in other T-ALL cases, combination therapies based on VEN have achieved great results [12].

A multicenter retrospective cohort study compared the efficacy of VEN combined with chemotherapy to VEN combined with Navitoclax (a novel BCL-XL inhibitor) and chemotherapy in 11 R/R-T-ALL patients. The results indicated that the objective response rates (ORR) for the two treatment regimens were 55% and 46%, respectively, suggesting that the addition of Navitoclax to chemotherapy may not significantly enhance efficacy. Also, it’s worth noting that the infections and liver toxicity were more common in the regimen that included Navitoclax [13]. However, in another study, 47 patients with R/R T-LBL/ALL achieved a 60% CR rate when treated with combination of VEN, Navitoclax and chemotherapy [14]. Also, VEN combined with larotrectinib demonstrated potential maintenance effects in a R/R ETV 6-NTRK3 positive T-ALL patient after allo-HSCT [15]. Recently, Summers et al. revealed that combination of MERTK and BCL-2 inhibitors may be effective for T-ALL/ETP-ALL using cell lines and xenograft models [16]. Roberta et al. reported that 3 R/R ETP-ALL patients experienced significant benefits with VEN combined bortezomib [17]. In another study involving five patients, all the patients achieved remission with VEN and azacitidine within one month of treatment, which included four patients with CR and one patient with incomplete recovery of blood cell counts (Cri). Among the four patients with CR, three achieved MRD negative CR (< 0.01%) [18]. Additionally, a patient with ETP-ALL with a TP53 mutation achieved MRD negative CR with a cycle of VEN and low-dose decitabine [19].

In a series of studies, the combination of VEN with chemotherapy has shown promising results in the treatment of T-ALL. Firstly, morphologic remission was attained in a 71-year-old woman with ETP-ALL using attenuated doses of hyper-CVAD (minus doxorubicin) alternating with methotrexate and cytarabine (mini-CVD) plus VEN [20]. Moreover, in another report, two patients with ETP-ALL achieved MRD negative CR with short-course VEN alongside standard chemotherapy [21]. Furthermore, a significant response was observed in a study where 3 out of 4 (75%) patients with R/R T-ALL achieved CR with mini-hyper-CVD plus VEN [22]. In a more comprehensive study involving 13 patients with T-cell disease, which included six cases of T-LBL, one case of ETP-ALL, and six cases of T-ALL), 77% (*n* = 10) patients achieved CR/Cri with VEN combined standard chemotherapy. Notably, among these, 8 out of 13 patients with R/R disease [23]. Additionally, a R/R T-ALL patient achieved CR with VEN, pegaspargase, and nelarabine, which served as a bridge to allo-HSCT [24]. In a separate case, an ETP-ALL patient initially received 2 cycles of VICP (vincristine, idarubicin, cyclophosphamide, and prednisone) and achieved MRD positive remission, and achieved MRD negative CR with one cycle of VEN plus CAG (aclacinomycin, cytarabine, and G-CSF) [25]. A retrospective study involving 13 patients, including 3 T-ALL/ETP-ALL patients, further confirmed that the treatment regimen combining VEN with other chemotherapy drugs showed good tolerability and safety in ALL patients with poor early response [26]. These findings collectively underscore the potential of VEN combined with chemotherapy in improving treatment outcomes for T-ALL, warranting further investigation into its role in the therapeutic landscape of these aggressive leukemia.

HHT, a recognized protein synthesis inhibitor, playing a significant role in the treatment of hematological diseases such as AML, CML, MDS, and polycythemia vera by regulating mechanisms including KIT, MYC, MCL-1 and Cyclin-D1 [27–29]. Recent studies have demonstrated that HHT can exert antitumor effects in T-ALL by inhibiting the NOTCH/MYC pathway, which has been shown to significantly prolong the survival of T-ALL mouse and patient-derived xenograft models [30]. Furthermore, basic research has revealed that HHT and VEN can synergistically induce the downregulation of the key anti-apoptotic protein MCL1, thereby enhancing apoptotic activity [31]. Despite these laboratory findings, clinical reports on the application of VEN in combined with HHT for T-ALL are scarce. One such report indicated that 6 patients with R/R ETP-ALL achieved encouraging efficacy with VEN-HAG regimen (VEN, Cytarabine, HHT, G-CSF) [31]. However, up to now, no similar reports on the applications of this combination in R/R-T-LBL/ALL outside of the ETP-ALL subset so far. In our study, V-CHG regimen was different from which reported by Suo S [31]. Here, we revised the previous V-CHG regimen and reduced the dosage and duration of drug. In the present study, the V-CHG regimen was as follows : VEN 100 mg d1, 200 mg d2, 400 mg d3, for 10–14 days, Ara-C 10mg/m^2^ q12h, subcutaneously, for 10–14 days, HHT 1mg/m^2^, continuously infused, for 10–14 days, and G-CSF 300 µg/d, subcutaneously, from day 0 until WBC count > 20 × 10^9^/L; In patients older than 60 years, the dose of HHT was reduced to 1 mg/d. If treatment response was achieved after 1 cycle, V-CHG regimen would be consolidated for one or two cycles. We observed a surprising response to V-CHG based regimen in the three non-ETP R/R-T-LBL/ALL cases. All the patients achieved CR with a cycle of V-CHG based regimen, with one patient achieving an MRD negative CR (< 1 × 10^−4^) after one cycle for up to 4 months until relapse again and another achieving an MRD negative CR (< 1 × 10^−6^) after two cycles for 11 months up to now. The patients experienced rapid remission, and the adverse events were tolerable. Therefore, our findings suggest that the V-CHG regimen is both effective and safe for R/R T-LBL/ALL patients.

It should be pointed out that this retrospective study also has some limitations. Given the old age and potential tolerability, the first patient (Case 1) received V-CHG regimen with only a daily dose of VEN for 100 mg, but not 400 mg. The latter two patients (Case 2 and 3) were treated with a regular daily dose of VEN for 400 mg. Due to the rapid progression to T-ALL (Case 2), we temporarily added two doses of bortezomib on the basis of V-CHG. Although VEN was effective in 3 R/R ETP-ALL patients combined with bortezomib, at least 4 doses of bortezomib was added in previous study [17]. Therefore, to our knowledge, bortezomib might not be the main reason for CR in our study. In future trial, we will use standard daily dose of VEN for 400 mg without adding bortezomib into V-CHG regimen. Previous study suggested VEN response correlated with higher bcl-2 expression in multiple myeloma [32]. Unfortunately, all the three patients in our study, pathological IHC and flow cytometry did not routinely label BCL-2. In the future, it is needed to observe the relationship between bcl-2 expression and V-CHG treatment response. Of note, considering only three patients in this study received the V-CHG regimen, thus it merits further exploration and application in the future clinical practice.

## Conclusion

In summary, our study has demonstrated that all the three patients with non-ETP R/R-T-LBL/ALL achieved good efficacy in the salvage regimen of V-CHG. However, it is important to acknowledge the limitations inherent in our study, specifically the small number of cases and some heterogeneity in patient characteristics. Consequently, to further validate these findings, a large cohort of cases must be collected.

## Data Availability

The datasets generated during and/or analyzed during the current study are available from the corresponding author on reasonable request.gzx2962@outlook.com.
